# Proteogenomic analysis of granulocyte macrophage colony- stimulating factor autoantibodies in the blood of a patient with autoimmune pulmonary alveolar proteinosis

**DOI:** 10.1038/s41598-020-61934-y

**Published:** 2020-03-18

**Authors:** Atsushi Hashimoto, Shiho Takeuchi, Ryo Kajita, Akira Yamagata, Ryota Kakui, Takahiro Tanaka, Koh Nakata

**Affiliations:** 1 Niigata University Medical & Dental Hospital, Niigata, Japan; 2 Niigata University Graduate School of Medical and Dental Sciences, Niigata, Japan; 3 Bruker Japan K.K., Kanagawa, Japan; 4 IDEA Consultants, Inc., Osaka, Japan

**Keywords:** Proteomics, Autoimmunity, Immunogenetics

## Abstract

Recently, attempts to reveal the structures of autoantibodies comprehensively using improved proteogenomics technology, have become popular. This technology identifies peptides in highly purified antibodies by using an Orbitrap device to compare spectra from liquid chromatography–tandem mass spectrometry against a cDNA database obtained through next-generation sequencing. In this study, we first analyzed granulocyte-macrophage colony-stimulating factor (GM-CSF) autoantibodies in a patient with autoimmune pulmonary alveolar proteinosis, using the trapped ion mobility spectrometry coupled with quadrupole time-of-flight (TIMS-TOF) instrument. The TIMS-TOF instrument identified peptides that partially matched sequences in up to 156 out of 162 cDNA clones. Complementarity-determining region 3 (CDR3) was fully and partially detected in nine and 132 clones, respectively. Moreover, we confirmed one unique framework region 4 (FR4) and at least three unique across CDR3 to FR4 peptides via *de novo* peptide sequencing. This new technology may thus permit the comprehensive identification of autoantibody structure.

## Introduction

Pulmonary alveolar proteinosis (PAP) is a rare lung disorder characterized by the accumulation of surfactant material in the alveoli and terminal bronchioli^1–3^.

PAP is classified into three different forms: autoimmune, congenital, and secondary^1–3^. Autoimmune PAP (aPAP) accounts for 91% of all PAP cases^1–3^. It is thought to be caused by granulocyte-macrophage colony-stimulating factor (GM-CSF) autoantibodies (GMAbs), which interfere with alveolar macrophage function and maturation, resulting in impaired surfactant catabolism. However, the mechanism involved in the excessive production of GMAbs remains unclear.

According to a previous study, GMAb is a polyclonal antibody with immunoglobulin M (IgM-), IgG-, and IgA-isotypes^4^. IgG-GMAb is pathogenic, but IgM-GMAb is thought to be a bystander etiologically, because its binding avidity to GM-CSF is 100-fold lower than that of IgG-GMAb, and its neutralizing capacity is extremely weak, i.e., it has a 20,000-fold higher IC_50_ than IgG-GMAb^4^. The currently available evidence regarding the effects of IgA-GMAb in the peripheral blood is limited, and its pathogenic role remains unclear.

Serum GMAbs are normally present in healthy individuals, although their concentration is much lower than that measured in patients with aPAP^5^. Our previous study have reported that circulating GM-CSF autoreactive B cells (GMARBs) producible of GMAb by Epstein-Barr virus transformation are consistently detected in healthy individuals and patients with aPAP^6^. These are potential precursor cells for GMAb-producing plasma cells and B cells.

Determining the relationship between the structures and functions of GMAbs is important for deepening our understanding of the pathogenesis and etiology of aPAP. To date, the structure of GMAbs has been investigated by analyzing monoclonal antibodies of clones isolated from patients’ B cells, which have been transformed using the Epstein–Barr virus^7^, or generated via single-cell polymerase chain reaction (PCR). Circulating GMARBs harboring surface GMAb for B-cell receptors were detected in peripheral blood samples from both healthy individuals and patients with aPAP^4^. Piccoli *et al*. (2015) established three non-cross-competing monoclonal GMAbs bound simultaneously to a single molecule of GM-CSF to form a high-molecular-weight immune complex, resulting in efficient *in vitro* neutralization of GM-CSF biological activity and promoted the rapid degradation of GM-CSF-containing immune complexes *in vivo* in an Fc-dependent manner^8^. Wang *et al*. generated 19 monoclonal GMAbs from six patients, these clones used multiple V genes, excluding preferred-V-gene use as an etiology, and targeted at least four non-overlapping epitopes on GM-CSF^9^. This suggested that it is GM-CSF that drives the production of autoantibodies, and not a B-cell epitope in a pathogen cross-reaction with GM-CSF^9^.

However, none of these studies could overcome the issue of selection bias. Creating a bias-free cDNA database of antibody sequences from sorted GMARBs using next-generation sequencers developed after 2007 would solve this problem. However, not all sorted cells are necessarily differentiated into plasma cells; thus, identifying the full range of antibodies in the sera of patients with aPAP using these technologies is challenging.

Proteogenomics technology identifies peptides produced from enzymatically digested proteins by comparing tandem mass spectrometry (MS/MS) spectra against a cDNA database that has been derived from next-generation sequencing^10^. This technology has been applied to the identification of sequences of monoclonal or polyclonal antibodies induced by antigen challenge in animals^11^. It identified 77% of the whole variable region sequence that was encoded in the cDNA database at the peptide/protein level for antigen-induced polyclonal antibodies^11^. Immune induction may render the identification of low-abundance regions, especially CDRs.

More recently, liquid chromatography coupled with MS/MS (LC/MS/MS) using an Orbitrap device (offering high peptide sensitivity and resolution) enabled studies to determine the sequence diversity of polyclonal autoantibodies in certain diseases. One study searched a cDNA database generated by next-generation sequencing and *de novo* peptide sequencing for mass spectral interpretation independent of a reference database^12^. This search identified, in the sera of patients with celiac disease, 20–75 clonotypes per patient containing the complex peptide sequence diversity necessary for IgA that reacts with a gluten-derived peptide or the autoantigen transglutaminase 2^12^. Using phage display combined with LC/MS/MS on an Orbitrap device, Chen *et al*. reported that the anti-desmoglein-1 or -3 autoantibodies in pemphigus were oligoclonal antibodies with less than 10 clonotypes^13^.

In this study, a new instrument offering a type of LC/MS/MS^14^ termed trapped ion mobility spectrometry coupled with quadrupole time-of-flight, the TIMS-TOF instrument, was used to identify trypsin-digested GMAb-derived peptides. In addition to accurate determination of the *m/z* in the TOF analyzer, TIMS offers separation based on size to charge (Ω/z) providing additional separation power and increased peak capacity. This is achieved by means of the parallel accumulation serial fragmentation (PASEF) method, which realizes a very high sequencing speed without a decrease in sensitivity and allows the selection of 100–350 precursors per second^14–16^.

The purpose of this study was to assess the analytical accuracy of TIMS-TOF instrument regarding the polyclonality of GMAb, by referring or not referring to a cDNA database created through genetic analysis.

## Results

### Creation of a GMARB cDNA database

**Figure 1:**
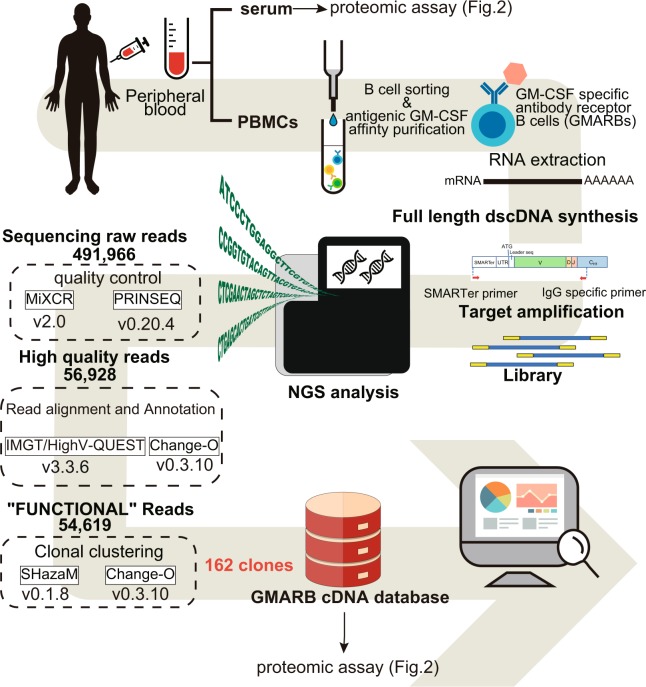
Basic workflow for the generation of full-length cDNA of GMARBs, followed by next-generation sequencing of immunoglobulin G heavy chain. PBMCs and serum were simultaneously extracted from whole blood (20 mL) obtained from a patient with aPAP. GMARBs were isolated, and the full-length cDNA library was generated. This was followed by the synthesis of bias-free PCR amplicons coding an IgG heavy chain variable region, and next-generation sequencing. The raw data were pre-processed by quality trimming using FASTX and applied to read alignment by IMGT High-V-QUEST, to identify the functional reads. Numbers in the dotted squares indicate the number of nucleotide sequence reads. Thereafter, classification of the functional reads into clones was performed using the clone clustering software Change-O. The methods used for clustering are described in the Online Methods.

Peripheral blood mononuclear cells (PBMCs) were purified from the patient’s blood and GMARBs were isolated and subjected to total RNA extraction (Fig. 1). From the total RNA, full-length cDNA with the SMARTer-oligo sequence at 5′ end was subjected to PCR to generate amplicons for variable regions of the IgG heavy chain (IgG-HV). Through 2 × 300 bp paired-end sequencing using an Illumina Miseq, 491,966 reads were obtained for the first raw reads, which were preprocessed to 56,928 high-quality reads using PRINSEQ version 0.20.4, followed by MiXCR Immune Repertoire Analyzer version 2.0. Subsequently, reads were assigned to the V (D) J germline segments of the Ig sequences for annotation using the international ImMunoGeneTics/HighV-QUERy and STandardization (IMGT/HighV-QUEST) tool^17^, resulting in 54,619 functional reads. These were further clustered to 162 clones, using the package Change-O to group clonotypes according to the V and J alleles and the nucleotide Hamming distance. These sequences from GMARBs were used to construct the reference databases and were used to analyze the LC/MS/MS spectra in the subsequent analysis.

### Characterization of the patient derived GM-CSF autoantibody

The GMAb showed high avidity to GM-CSF with Kd at 20.8 pM and a strong neutralizing capacity against recombinant *E. coli*-derived GM-CSF (0.5 ng/mL) with IC_50_ value of 64.5 ng/mL. From 6 ml of the serum 185 μg of GMAb with IgG isotype was obtained. The purified GMAb was applied to 2D-PAGE analysis (see Supplemental Fig. S1) and found to contain all subclass of IgG (see Supplemental Fig. S2).

### Analysis of highly purified circulating GMAb via high-resolution LC/MS/MS and quadrupole TOF

**Figure 2:**
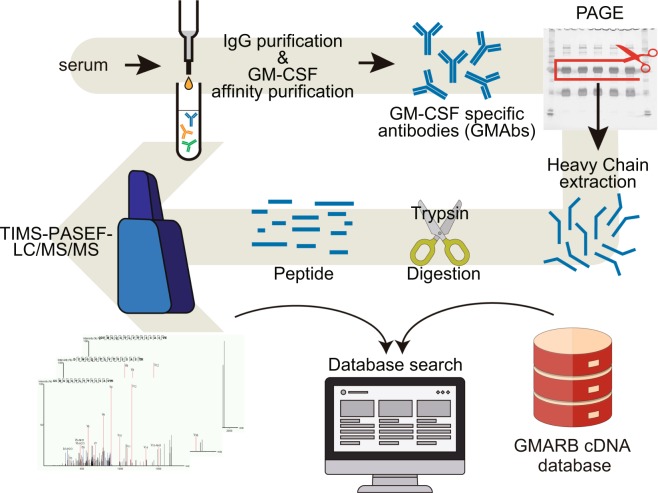
Proteomic platform for the identification of peptides derived from purified GM-CSF autoantibodies (GMAbs). Serum from a patient was processed for the purification of IgG, followed by isolation of GM-CSF autoantibodies using a GM-CSF-coupled column. Isolated GMAbs were loaded on SDS-PAGE. The band for the IgG-heavy chain was cut and subjected to in-gel digestion using trypsin. The resultant peptides were purified with a C18 column and subjected to nano-elute UH-PLC-coupled TIMS-TOF instrument MS. Raw MS/MS data were subjected to analysis using PEAKS Studio 8.5 for data processing.

**Figure 3:**
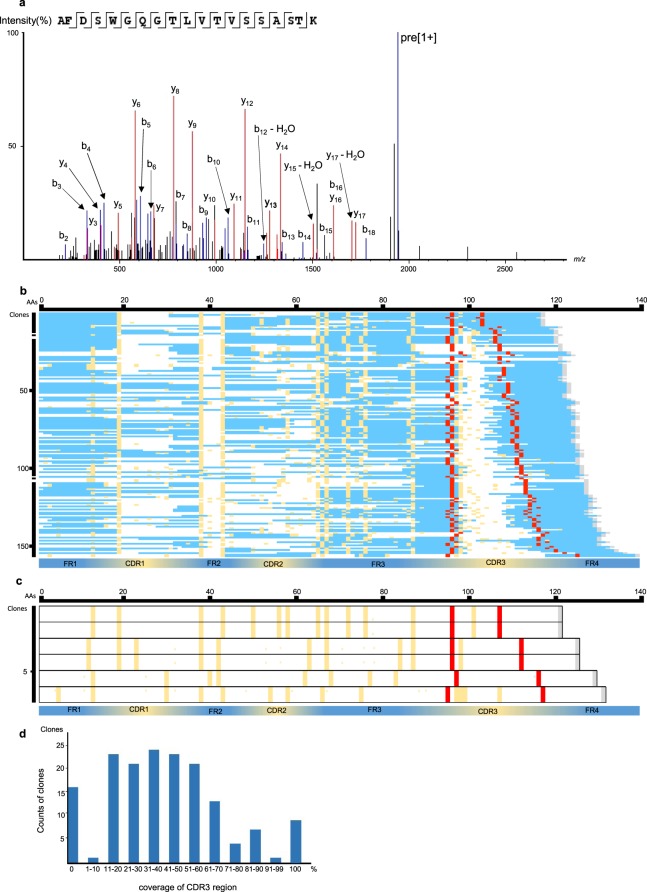
Identification of peptide sequences coded in clones listed in the cDNA database using a peptide sequence analysis software-filtered peptide sequences. (**a**) An example of MS/MS spectrum matched by the software to the V-region full tryptic peptide AFDSWGQGTLVTVSSASTK containing CDR3 (m/z = 971.4794, z = 2, RT = 73.24, ppm = −1.9). (**b**) Coverage of 156 cDNA clones partially matching the PEAKS-filtered peptides. The peptides in which the amino acids were covered are shown as blue bars. The red bars indicate CDR3 anchor amino acids. The yellow bars indicate candidates for trypsin cleavage sites (i.e., arginine or lysine). The gray bars indicate the end of an amino acid. The vertical and horizontal axes indicate the number of clones and the amino acid positions from the N-terminal end, respectively. (**c**) Amino acid sequences deduced from the six cDNA clones of which coverage by the software-filtered peptides was zero. (**d**) The number of clones with different amounts of coverage by the software-filtered peptides in the CDR3 region. The vertical and horizontal axes indicate the number of clones and the coverage, respectively.

The peptide mixture derived from the highly purified GMAb was subjected to an ultra-high-performance LC coupled online with a hybrid TIMS-TOF instrument with an ion source (Fig. 2). The filtered result through a peptide sequence analysis software matched 4,000 spectra with 827 peptide sequences. An example of mass spectrum was shown in Fig. 3a. The 827 peptides shared some sequence similarity with IgG-HV in up to 156 out of the 162 clones listed in the cDNA database (Table 1, Fig. 3b). Overall, median of no less than 63.4% [50.4%, 79.3%] of the total amino acid sequences were covered. Coverage appeared relatively lower for the CDR1 and CDR2 regions (20% [0%,87.5%] and 0% [0%, 100%], respectively), whereas it was high for framework region 1 (FR1) (76% [0%, 82%]) and FR4 (100% [100%, 100%]). In particular, a peptide (amino acid position of around 39–43 amino acids) – a region of five amino acids flanked by two tryptic cleavage sites – was hardly covered in 140 of the 156 clones. Six clones that were not covered by the software-filtered result at all exhibited a similar structure in terms of the putative peptide length of FRs and CDRs by tryptic digestion and candidate position of tryptic cleavage sites (Fig. 3c).

**Table 1:** Identification of IgG-HV clones by TIMS-TOF instrument.

	Identification type	Clones
Clones identified by TIMS-TOF instrument [total]		156
	Clones with fully identified CDR3	9
	Clones with partially identified CDR3	132
	Clones with non-identified CDR3	15
Clones not identified by TIMS-TOF instrument		6

IgG-HV were identified by TIMS-TOF instrument followed by a peptide sequence analysis software search.

Count of clones with CDR3 identified based on their peptides by TIMS-TOF instrument in the cDNA database.

TIMS-TOF instrument: trapped ion mobility spectrometry coupled with quadrupole time-of-flight mass spectrometry.

The software-filtered result covered the full length of complementarity-determining region 3 (CDR3) in nine of the 156 clones, partially covered CDR3 in 132 of the clones, and did not cover it at all in 15, giving a median coverage of 37.6% ([20.2%, 54.5%]; Table 1, Fig. 3d). There was a weak correlation between the length of the CDR3 region and the rate of coverage by the software-filtered result (ρ = −0.36, p < 0.001); however, the number and position of putative tryptic cleavage site (K and R) did not affect coverage. In the nine clones in which the whole sequence was covered, the CDR3 region comprised <10 amino acids. The second half of the FR3 had a relatively high coverage rate and the peptide consistently ended at the first tryptic cleavage site, near the beginning of the CDR3 region. In contrast, the beginning of the peptides leading to the FR4 region appeared to not be affected by the occurrence of tryptic cleavage sites located inside the CDR3 region. The diversity of IgG-HV was also confirmed by 2 dimensional development combined with TIMS-TOF followed by the software search (See Supplementary Result).

### *De novo* CDR3-FR4 analysis

**Figure 4:**
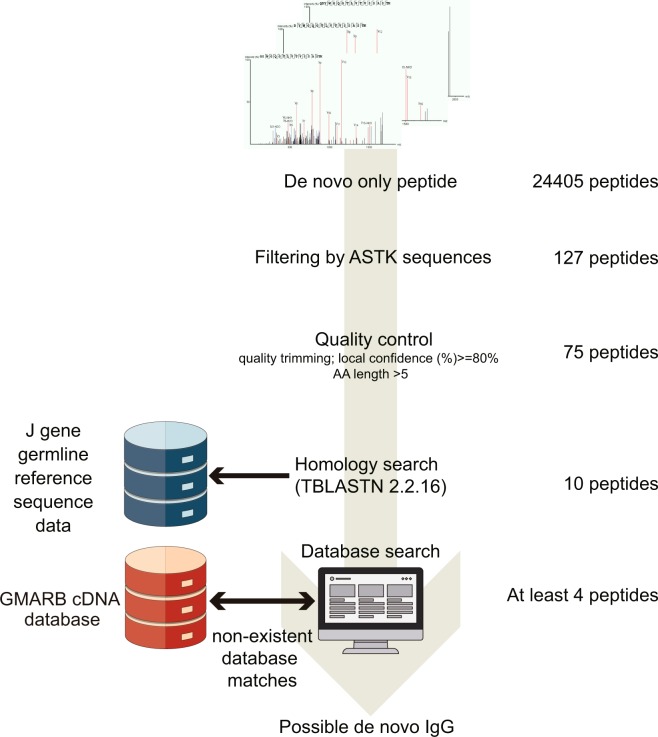
Procedures for the *de novo* peptide sequencing of the CDR3–FR4 region. From the peptides unidentified by a peptide sequence analysis software search, those containing “ASTK” sequences were extracted. Seventy-five peptides with local confidence <80% were excluded. The peptides were further selected via a homology search for J gene reference sequence data; four of them were not listed in the cDNA database.

We conducted *de novo* peptide sequence estimation from raw LC/MS/MS spectra not listed in the cDNA database to determine whether the patient’s serum contained any GMAb clones other than those coded in the cDNA database. A total of 24,405 peptides remained unidentified by the software database search algorithms and are thus putative *de novo* sequences. Of these, 127 contained the “ASTK” sequence at the C terminal end, which is characteristic of the beginning of the constant region of the IgG isotype. The software assigns a local confidence score for each amino acid in a *de novo* sequence. The local confidence score ranges from 0% to 99%, indicating the confidence of the algorithm that a particular amino acid is correctly sequenced. Low-scoring amino acid (i.e., amino acid with a local confidence score of <80%) were trimmed, and short-length peptides (<5 amino acids) were excluded. Seventy-five peptides were further selected as quality control, as described in the Methods section (see Online Methods, Fig. 4). Of these, 10 matched the J gene sequence when we searched for homology with J gene reference sequence data (TBLASTIN 2.2.16). One and at least three clones were confirmed to have *de novo* sequence of FR4 and across CDR3 to FR4 regions, respectively, from the cDNA database (Table 2, Supplemental Table 1).

**Table 2:** CDR3–FR4 sequences identified by *de novo* peptide sequencing.

No.	Qualified peptides	TBLASTN_hit	FR4_matched	CDR3_matched	Putative *de novo*
1	AHGSSTDH**W**GQGTLVTVSSAS	Yes	Yes	No	Yes
2	AHGSSESH**W**GQGTLVTVSSAS	Yes	Yes	No	Yes
3	H**W**GQGTLVTVSSAST	Yes	Yes	No	(Yes)
4	V**W**GQGTTVTVSSAS	Yes	Yes	N/A	
5	DVP**W**GQGTLVTVSS	Yes	Yes	No	Yes
6	L**W**GLGTLVTVSSA	Yes	No	N/A	Yes
7	Y**W**GQGTLVTVSS	Yes	Yes	N/A	
8	**W**GQGTLVTVSS	Yes	Yes	N/A	
9	Y**W**GQGTPVTVS	Yes	N/A	N/A	
10	**W**GQGTLVTVS	Yes	N/A	N/A	

N/A: not applicable.

(Yes): “unclassified due to insufficient CDR3 length”.

The local confidence of each amino acid constituting the peptide was ≥80%. VH sequences in the CDR3 region and CDR3 anchor amino acids are indicated in black underlined and bold characters, respectively. “TBLASTN_hit” indicates whether the J gene germline reference sequence search via a TBLASTN homology search found a match. “FR4_matched” indicates whether the flag for the FR4 region in each peptide matched the FR4 region in the cDNA database. “CDR3_matched” indicates whether the flag for the CDR3 region in each peptide matched the CDR3 region in the cDNA database. “Putative *de novo*” indicates whether the peptide is a putative *de novo* peptide.

Abbreviations used in this article:.

aPAP, autoimmune PAP.

CDR, complementarity determining region.

FR, framework region.

GM-CSF, granulocyte-macrophage colony-stimulating factor.

GMAb, GM-CSF autoantibody.

GMARBs, GM-CSF autoreactive B cells.

IgG, immunoglobulin G.

IgG-HV, variable region of immunoglobulin G heavy chain.

IMGT, the international ImMunoGeneTics information system.

LC/MS/MS, liquid chromatography coupled with tandem mass spectrometry.

PAP, pulmonary alveolar proteinosis.

PBMCs, peripheral blood mononuclear cells.

PCR, polymerase chain reaction.

TBS, tris-buffered saline.

TIMS-TOF, trapped ion mobility spectrometry coupled with quadrupole time-of-flight mass spectrometry.

## Discussion

Complete inhibition of lung GM-CSF bioactivity by neutralizing GMAbs is critical for developing PAP. A recent study demonstrated that combination of multiple high affinity monoclonal anti-GM-CSF antibodies elicits strong neutralizing capacity in patients with aPAP^8^. To better understand the mechanism for generation of such strong antibodies, it is clue to know how many clones the patient’s GMAbs consist of and how the structure is diverse. For these purposes, we investigated the protein structure of the heavy chain variable region of GMAbs with high avidity and strong neutralizing capacity as described in “Results” session. We used proteome analysis by the newly developed LC/MS/MS TIMS-TOF instrument followed by a peptide sequence analysis software search to identify 156 of the total 162 clones listed in the IgG-HV cDNA database that matched enzymatically digested GMAb-derived peptides. This was supported by the fact that matched peptides were even identified for the hypervariable CDR3 region in 133 clones. The results suggested that most cDNA in GMARBs should actually be expressed as GMAb in serum. The results of the *de novo* peptide sequencing also suggested that some clones other than those in the cDNA database may be present in the patient’s serum.

Proteomic analysis of patient-derived autoantibodies often encounters difficulties. Regions other than CDRs have a high sequence homology among clones, so it is difficult to determine clone-specific sequences, whereas CDRs are highly variable and the number of peptides in each clone is insufficient for detection by LC/MS/MS. A high-performance mass analyzer with high sensitivity and resolution is thus required to overcome this limitation. To date, a number of studies involving proteomic analysis of antibody structure have used an Orbitrap device that traps ions in electrostatic fields and converts them to a mass spectrum using a Fourier transform of the frequency signal^18^. In contrast, this study used a novel instrument that combines LC/MS/MS with trapped ion mobility spectrometry, which offers additional separation power and increased peak capacity over instruments that do not perform trapped ion mobility separation^14,16^. There is little information available regarding comparison in performance between Orbitrap devices and TIMS-TOF instrument. One study that has compared their performance is that of Singhal *et al*., who performed a proteomic analysis of proteins extracted from paraffin-embedded sections. Using TIMS-TOF instrument with PASEF, they observed increased peptide and protein depth, due at least in part to the sensitivity and speed of the instrument: five times peptide than those identified by Orbitrap device^19^.

To investigate the structure of GMAb clones comprehensively using proteogenomics technology, it is necessary to prepare a bias-free IgG-HV cDNA database from circulating GMARBs. For this purpose, we applied the 5′ rapid amplification of cDNA ends (RACE) method^20,21^ to total RNA extracted from the patient’s GMARBs to generate a library of the full-length cDNA in the cells. We also applied high-throughput paired-end sequencing to maximize the number of reads obtained. Using pre- and postprocessing procedures, we succeeded in narrowing a sequence of approximately 500,000 reads down to 162 clones of antibody genes, using Change-O software to distinguish variations due to real mutations from those induced by PCR errors^22^.

However, the cDNA database itself does not necessarily reflect the presence of whole antibodies, because the 162 clones may not always differentiate into antibody-producing cells. Therefore, we investigated the existence of peptides deduced from the cDNA database using TIMS-TOF instrument followed by the software search. Most of the clones in the cDNA database reflected the variety of GMAbs in the serum, except for six that did not match any peptide assessed by the proteomic analysis. These six clones exhibited similar structural characteristics (e.g., regional length and tryptic cleavage sites) to the other 156 clones. They were therefore derived from memory B cells that may have been on the way to differentiating into GMAb-producing plasma cells. In contrast, *de novo* peptide sequencing confirmed that four clones may have differentiated into plasma cells in the past and did not currently exist in GMARBs.

In the present study the coverage of the peptides identified by TIMS-TOF instrument analysis differed markedly among the IgG-HV regions. The coverage of the FR4 region of 136 of the 162 clones was 100%, whereas the small peptide area in the FR2 between the two tryptic cleavage sites was not covered at all for 128 of the clones. This difference in coverage can be attributed to the characteristics of peptide identification by LC/MS/MS. Firstly, the peptide lengths that are likely to be detected by LC/MS/MS are 6–16 amino acids^23,24^. Secondly, the quantity of certain peptides is a critical factor for detection (i.e., whether low quantities can be detected or not depends on the sensitivity of LC/MS/MS). Thirdly, the presence of acidic or hydrophilic amino acids in the peptide interferes with its detection. Considering these characteristics, it makes sense that the median coverage was as high as 100% for the FR4 region, because it is highly homologous and contains few acidic and/or hydrophilic amino acids. Even though amino acid sequences are highly conserved, but when the cleaved peptide is excessively short, detection of peptides was difficult as demonstrated by the narrow area in the FR2 region.

Of the regions in IgG-HV, the CDR3 region is especially important because it is the most hypervariable region and is responsible for antigen specificity, as well as being the primary determinant of clonality. However, previous studies using Orbitrap nano-electrospray ionization tandem mass spectrometry demonstrated consistently low coverage of this region^25^. In contrast, the CDR3 region was at least partially covered in 132 of the clones in this study, with a median coverage of 37.6%. This is conclusive because high sequencing speed with PASEF and additional TIMS separation can improve CDR3 peptide identifications if *m/z* is similar. This suggests that proteomic analysis of autoantibodies using the TIMS-TOF instrument has even more potential if pretreatment methods, such as enzymatic digestion of autoantibodies, or combined analyses using several different enzymes are improved^11^.

By *de novo* peptide sequencing using the software-filtered result, we confirmed one unique FR4 and at least three unique across CDR3 to FR4 sequences that were not listed in the cDNA database. It is important to note that these numbers are minimum estimates. Seventy-five sequences were candidates for *de novo* sequences. However, most of these had <80% local confidence for any amino acid in the sequence, so the true number of *de novo* sequences was unclear. Regardless of the true number, it is rather important that some clones with *de novo* peptide sequences, not found encoded in the cDNA database, were found. This suggests that the GMARB population changes over time and that plasma cells may remain and continue to produce GMAb, even after the differentiation of the original memory B cells into plasma cells.

In conclusion, using the TIMS-TOF instrument, trypsin-digested peptides of GMAbs partially matched the sequences of 156 of 162 cDNA clones of GMARBs generated by genetic analysis, suggesting that most GMARBs differentiate into plasma cells to generate GMAbs in patients with aPAP. Proteomic analysis of divergent polyclonal antibodies, especially the CDR3 region, is difficult. However, the fact that TIMS-TOF instrument identified 81 sequences in the CDR3 region may be attributed to the high sensitivity of this system. We expect that this study will promote proteomic analysis of autoantibodies.

## Methods

### Subject

In 2004, an inherently healthy male aged 44 years became aware of shortness of breath on exertion. High-resolution computed tomography revealed the presence of an abnormal chest shadow. The patient was diagnosed with aPAP following bronchoalveolar lavage and serological testing at a local hospital. The patient’s serum was positive for GMAb. The study was approved by The Institutional Review Committee of Niigata University (No. 2015–2388). In addition to this, we confirmed that all methods were performed in accordance with the relevant guidelines and regulations. Statement on the guidelines followed the Declaration of Helsinki. Written informed consent was provided by the patient prior to enrollment in the study.

### Binding avidity assay

The avidity of GMAb levels was estimated as previously reported^26^. Diluted serum that contained 200 ng/ml of IgG-GMAb was incubated with biotinylated rhGM-CSF (0–50 ng/ml) at 4 °C for 1 h, transferred into a 96-well microtiter plate previously coated with anti-human IgG (1 μg/ml, Bethyl Laboratories, Montgomery, TX, USA). Biotinylated rhGM-CSF was added followed by reaction with streptavidin-HRP (Bethyl Laboratories) and detected with TMB One Component Substrate (Bethyl Laboratorie) using a microplate reader, (Bio-Rad, CA, USA). The Binding avidity was estimated using Lineweaver-Burk plot.

### Serum neutralizing capacity against GM-CSF

Neutralizing capacity of GMAb was evaluated using TF-1 cells as described previously^4,27^. Briefly, TF-1 cells (40,000 cells/well) were incubated at 37 °C under 5% CO_2_ in a flat bottom microtiter plates for 3 days in Macrophage Serum Free Medium (Thermo Fisher Scientific, Waltham, MA, USA) containing recombinant human GM-CSF (0.5 ng/ml) with various concentrations of GMAb (0–125 ng/ml). TF-1 cells survival was evaluated as formazan formation using the Cell Counting Kit-8 (Doujindo, Kumamoto, Japan), measuring the absorbance at 450 nm by using a microplate reader (Bio-Rad).

### Isolation of GMARBs from patient blood

Peripheral blood mononuclear cells (0.9 × 10^7^ cells) were isolated by density gradient centrifugation with Ficoll-Paque PLUS (GE Healthcare, Chicago, Illinois, USA), and B cells (0.4 × 10^5^ cells) were isolated using a Pan B cell isolation kit (Miltenyi Biotec, Bergisch Gladbach, Germany). GMARB were further purified by incubating with 500 ng/ml of biotinylated rhGM-CSF for 30 min on ice, followed by incubation with Anti-Biotin MicroBeads (Miltenyi Biotec) for 15 min on ice. The cells were applied to a MACS MS column (Miltenyi Biotec) and GMARB were collected according to the manufacturer’s instructions. Approximately 1.25 × 10^4^ GMARB were isolated.

### Preparation of full-length cDNA extracted from GMARBs

Total RNA was extracted from the isolated GMARBs using the RNeasy Micro kit (QIAGEN, Venlo, Netherlands) according to the instructions provided by the manufacturer. The RNA was reverse-transcribed into full-length cDNA using the SMARTer cDNA Synthesis Kit (Takara Bio, Shiga, Japan) to avoid amplification bias related to primer variability. Briefly, the modified oligo (dT) primer 3′ SMART CDS Primer II A (Takara Bio) was used for the first-strand single-strand cDNA synthesis reaction. When SMART Scribe Reverse Transcriptase (Takara Bio) reaches the 5′ end of the mRNA, the enzyme’s terminal transferase activity adds a few nucleotides to the 3′ end of the single-strand cDNA. The SMARTer Oligonucleotide base pairs with a non-template nucleotide stretch are added, creating an extended template. SMART Scribe Reverse Transcriptase switches the templates and continues replicating to the end of the oligonucleotide^21,28,29^. Double-strand cDNA was then synthesized using the Advantage 2 PCR Enzyme kit (Takara Bio).

### Preparation of a library of IgGH variable regions for use on next-generation sequencing platforms

The first PCR amplification for variable regions of the H chain was performed using KOD-Plus-Neo DNA polymerase (Toyobo, Osaka, Japan) with a SMARTer primer for the common 5′ primer and 3′ primer. The amplicons were purified and subjected to high-throughput 2 × 300 bp paired-end sequencing using the MiSeq v3 reagent kit (Illumina) with a 50% PhiX spike on the Illumina MiSeq platform, according to the recommendations provided by the manufacturer.

### Pre-processing of raw reads obtained through high-throughput sequencing

Quality control of raw reads obtained by high-throughput sequencing was performed as follows. For the H chain, the 3′ low-quality bases were trimmed; total sequences with lengths of <100 nucleotides were filtered out using paired-end reads, as were raw reads with a mean Phred quality score of <30. These steps were conducted using the quality control and data preprocessing functions of PRINSEQ (version 0.20.4)^30^. The two paired-end reads were subsequently assembled into a complete Ig sequence, the forward and reverse primers were removed, and the data were de-multiplexed for each isotype using the MiXCR Immune Repertoire Analyzer (version 2.0)^31^. The software assigned the reads to regions while discriminating between IgM and IgG, using the reference database (GenBank). Variable region sequence reads with incomplete lengths, unassembled read sequences, or read sequences with missing primers were also removed.

### V (D) J assignment and annotation

After quality control, reads were assigned to the V (D) J germline segments of the Ig sequences for annotation using IMGT/HighV-QUEST software (http://imgt.org, v1.5.0)^17^ and the IMGT gene database (version: 7 January 2016).

### Additional quality control and clonal clustering

For post-processing of the IMGT/HighV-QUEST output, additional quality control and clonal clustering were performed using several software programs, Change-O (version 0.3.10)^22^,and SHazaM (version 0.1.8)^22,32^, and custom scripts within the R statistical computing environment (version 3.3.3).

### Autoantibody purification

Serum (6 mL) from the same patient collected on the same day as described above was filtered with a 0.2 μm filter, diluted 8-fold with tris-buffered saline (TBS) (pH 7.4), and processed using a HiTrap rProtein A FF column (1 mL of resin). After washing with TBS, the IgG proteins were eluted with 4 mL of 100 mM glycine-hydrochloride (glycine-HCl) (pH 2.5), followed by neutralization with 1 M Tris-HCl (pH 8.6), dialysis against TBS for 2 d at 4 °C, and application to a GM-CSF-coupled HiTrap NHS-activated HP column.

After washing with 10 mL of TBS, nonspecifically bound IgG was further washed out via the addition of 10 mL of 50 mM ammonium acetate (pH 5.0). The IgG proteins binding to GM-CSF were eluted with 100 mM glycine-HCl (pH 2.5). The eluate was neutralized by adding 1 M Tris-HCl (pH 8.6), concentrated with Ultrafree-0.5 (5 kDa molecular weight cutoff) at 15,000 × g for 30 min, and loaded into a 10% sodium dodecyl sulfate-polyacrylamide gel for electrophoresis (Fig. 2).

### In-gel digestion and mass spectrometric identification of proteins

Protein bands were excised from the electrophoretic gels and dehydrated in acetonitrile. The gel pieces were rehydrated in a digestion solution consisting of 50 mM ammonium bicarbonate and 0.01 µg/µl modified sequence-grade trypsin (Promega, Madison, WI, USA)^33,34^. After overnight incubation at 37 °C, the digested peptides were extracted using 1% trifluoroacetic acid in 60% acetonitrile, and the extracted peptides were dried in a vacuum centrifuge^35–37^. The peptides were purified with ZipTip (Millipore, Burlington, MA, USA) according to the protocol provided by the manufacturer.

### LC/MS/MS analysis

The peptide mixture was loaded onto a C18 column (25 cm × 75 μm, 1.6 µm; IonOpticks, Melbourne, Australia). The mobile phases consisted of (A) 0.1% formic acid and (B) acetonitrile with 0.1% formic acid (volume per volume). The nano-flow LC gradient was delivered at 400 nL/min and consisted of a linear gradient of mobile phase B increasing from 2% to 17% B in 60 min, followed by increases to 25% B in 30 min, 37% in 10 min, and 95% B in 10 min. Ions were collected in the TIMS device over 100 ms and MS and MS/MS data were acquired over an *m/z* range of 100–1,700. During the collection of MS/MS data, the TIMS cycle was adjusted to 1.1 s and included 1 MS plus 10 PASEF-MS/MS scans, each containing on average 12 MS/MS spectra (>100 Hz)^15,16^.

### cDNA database search and data processing

All raw LC/MS/MS data were submitted to PEAKS Studio 8.5 (Bioinformatics Solutions, Waterloo, Canada) for data processing^38^. The cDNA database described above was used as a reference, and a decoy database was constructed for use in this analysis to control false discovery rates^39^. Search parameters included trypsin as the enzyme, with up to one missed cleavage allowed. Carbamidomethylation of cysteine residues was set as a fixed modification, and oxidation of methionine was set as a variable modification. Parent mass error tolerance was set to 10 ppm, while fragment mass error tolerance was set to 0.05 Da. False discovery rates were adjusted to 1% at the peptide spectrum matches.

### *De novo* peptide sequencing

*De novo* sequencing^40^ was used to identify peptides harboring the CDR3–FR4 regions, characterized as amino acid sequences ending in ASTK (IgG)^12^. The list of exclusively *de novo* peptides includes high-quality peptide sequences detected by *de novo* sequencing that remain unidentified by the PEAKS database search. The peptides containing ASTK sequences were extracted from this list. Low-quality amino acids (local confidence <80%) were trimmed. Peptide sequences with lengths of less than five peptides were excluded. The novel proteins that required *de novo* sequencing and lacked similar proteins (for the CDR3–FR4 regions) in our cDNA databases were defined via the following two steps. Firstly, the datasets were searched against the J gene reference sequences obtained from the IMGT/LIGM-DB reference sequences database to identify the J gene. Next, a second search was performed against the in-house cDNA next-generation sequencing database assembled for the identified CDR3–FR4 region sequence.

### Statistical analysis

Non-normally distributed data are reported as medians with interquartile [25%, 75%]. Spearman’s correlation coefficient was used to estimate the relationship between two parameters. Statistical analyses were performed on a microcomputer using JMP (12.0.1) software (SAS Institute, Cary, NC, USA).

## Data Availability

NGS data that support the findings of this study have been deposited in the DDBJ Japanese Genotype-phenotype Archive (JGA, http://trace.ddbj.nig.ac.jp/jga)^41^ with the accession codes JGAS00000000163. The mass spectrometry data have been deposited to the ProteomeXchange Consortium (http://proteomecentral.proteomexchange.org) via the jPOSTrepo (https://repository.jpostdb.org/)^42^ with the accession numbers PXD015420 /JPST000674.

## Supplemental Information

Supplemental Information.
Supplemental Information.
