# Supplementary material for: Proteogenomic analysis of granulocyte macrophage colony- stimulating factor autoantibodies in the blood of a patient with autoimmune pulmonary alveolar proteinosis
Source: Sci Rep. 2020 Mar 18;10:4923. doi: 10.1038/s41598-020-61934-y (PMC7080758; doi:10.1038/s41598-020-61934-y)
Supplement: Supplementary file 1 — Supplemental Information. [file 41598_2020_61934_MOESM1_ESM.pdf]

Supplementary information

Proteogenomic analysis of granulocyte macrophage colony- stimulating factor autoantibodies in the blood of a patient with autoimmune pulmonary alveolar proteinosis

Atsushi Hashimoto[1†], Shiho Takeuchi [2†], Ryo Kajita[3], Akira Yamagata[4], Ryota Kakui[4], Takahiro Tanaka[1], and Koh Nakata[1*]

[1] Niigata University Medical & Dental Hospital, Niigata, Japan

[2] Niigata University Graduate School of Medical and Dental Sciences, Niigata, Japan

[3] Bruker Japan K.K.

[4] IDEA Consultants, Inc., Osaka, Japan

† These authors contributed equally.

*Corresponding author: Koh Nakata, MD, PhD

E-mail address: radical@med.niigata-u.ac.jp

Supplementary Result

Identification of proteins in two-dimensional (2D) spots separated from highly purified and deglycosylated GM-CSF autoantibody.

Affinity purified polyclonal GMAbs IgG from a patient with aPAP (50 micrograms) was subjected to 2D-PAGE and proteins visualized by fluorescent staining as described in Supplementary Methods. As shown in Supplementary Figure S1, approximately 19 individual spots at around 50 KDa corresponding to the heavy chain with pI ranging 6 to 9.5 appeared in the 2D development. Trypsin-digested each spot peptides were analyzed by LC/MS/MS TIMS-TOF instrument followed by a peptide sequence analysis software search. This allowed an extensive characterization of both constant and variable regions. Coverage of the constant region was consistently high, whereas it was low in the variable region. Fifty two different peptides were identified in the whole spots, which matched at least some part of variable region of clones listed in cDNA database. Only 17 peptides of them were identified to be unique peptides in 11 spots (Supplementary Figure S2a). That corresponded to a single clone in the cDNA database. Since the other 35 peptides matched variable region

sequences common to multiple cDNA clones, for such peptides, involved spot number could not correspond to cDNA clone number in one to one fashion. Thus, each identified peptide likely corresponded to multiple cDNA clones. As the subtypes of IgG were not clearly segregated by pI (Supplementary Figure S2b), the diverse physicochemical properties of spot proteins was likely due to variety of peptides pI involved in the variable region.

These results suggested that the diversity GMAb clones was demonstrated by not only proteogenomic analysis but also 2D development.

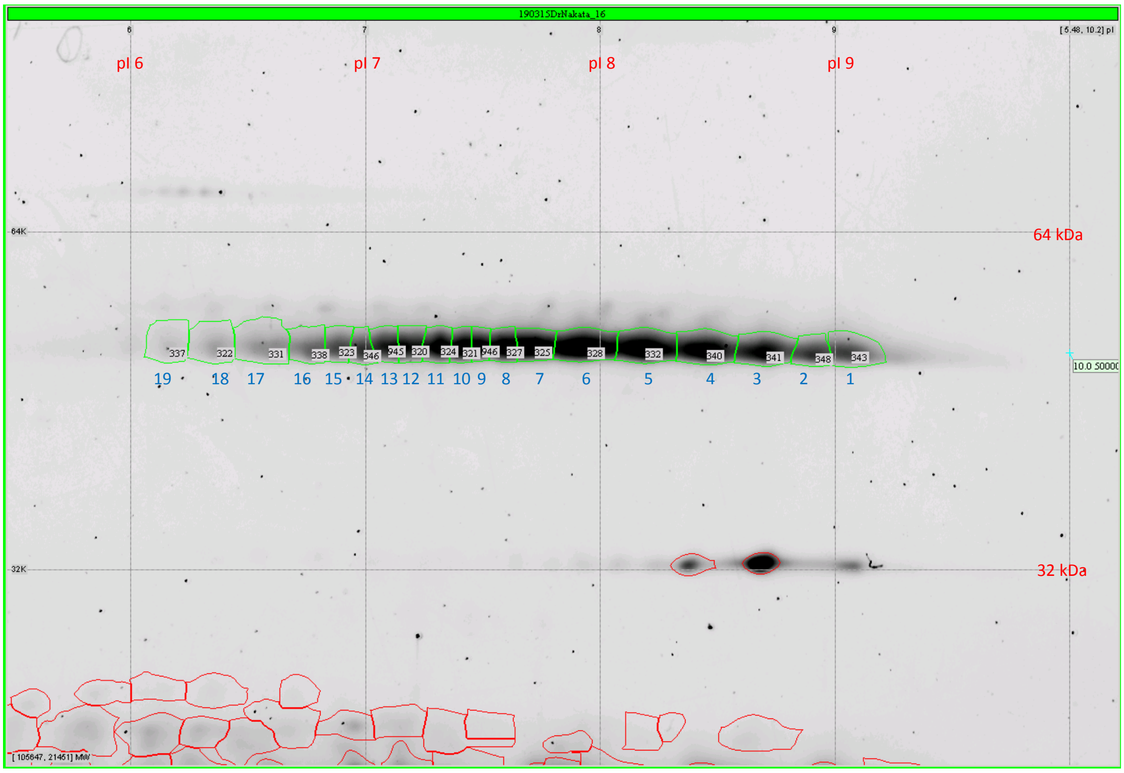

Supplementary Figure S1

2-D gel separation of affinity purified polyclonal GMAbs IgG

Vertical and horizontal indicates isoelectric point (pI) and molecular weight (kDa), respectively. Spots around 50 kDa were surrounded by green column with spot numbers. Nineteen spots were excised, digested, and analyzed by TIMS-TOF instrument.

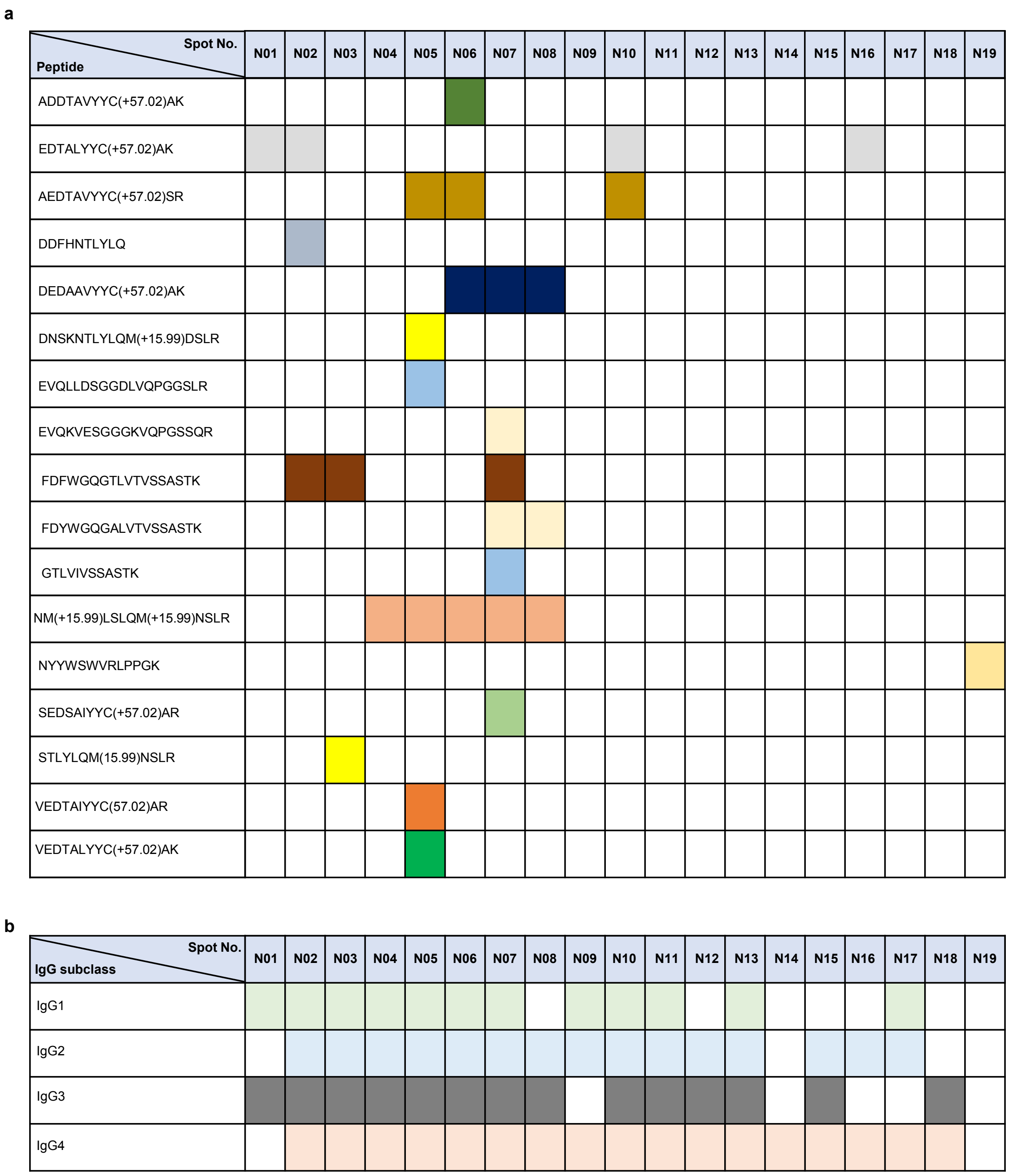

Supplementary Figure S2

The distribution map of the unique peptides of variable (a) and constant (b) regions identified in 2DE spots separated from GM-CSF autoantibody.

(a) The unique peptides assigned to variable region of heavy chain. The unique peptide associated with a single clone in a spot. Each clone was distinguished by colors. (b) The possibility of four IgG subclass exists in each spot.

Supplementary methods

Two-dimensional gel electrophoresis

The purified antibody (20 micrograms) was subjected to sugar chain removal treatment by a PNGase F PRIME Glycosidase（N-Zyme Scientifics LLC, Doylestown, PA）. Protein samples after glycan removal were applied to Immobiline Drystrip (pI 3-10, 18 cm, GE Healthcare UK Ltd, Buckinghamshire, England) by in-gel rehydration[1,2]. The isoelectric focusing was performed in a Multiphor II electrophoresis unit (GE Healthcare) according to the manufacturer's instruction. As the second dimension, SDS-PAGE performed in 9-18% acrylamide gradient gels using an IsoDalt electrophoresis system. The 2-D gels were stained with SYPRO Ruby (Thermo Fisher Scientific, Waltham MA. USA). The protein spots were detected using a Molecular Imager FX (Bio-Rad Laboratories, Hercules CA. USA) and analyzed using ImageMaster 2D Platinum (GE Healthcare)[3,4].

References

1. Sanchez, J.-C. *et al.* Improved and simplified in-gel sample application using. *Electrophoresis* **18**, 324–327 (1997).
2. Rabilloud T, Valette C, L. J. Sample application by in-gel rehydration improves the resolution of two-dimensional electrophoresis with immobilized pH gradients in the first dimension..pdf. *Electrophoresis* **15**, 1552–8 (1994).
3. Kristensen, D. B. *et al.* Mass spectrometric approaches for the characterization of proteins on a hybrid quadrupole time-of-flight (Q-TOF) mass spectrometer Proteomics and 2-DE. *Electrophoresis* **21**, 430–439 (2000).
4. Lopez, M. F. *et al.* A comparison of silver stain and SYPRO Ruby Protein Gel Stain with respect to protein detection in two-dimensional gels and identification by peptide mass profiling. *Electrophoresis* **21**, 3673–3683 (2000).
